# Safety, Pharmacokinetic, and Efficacy Studies of Oral DB868 in a First Stage Vervet Monkey Model of Human African Trypanosomiasis

**DOI:** 10.1371/journal.pntd.0002230

**Published:** 2013-06-06

**Authors:** John K. Thuita, Kristina K. Wolf, Grace A. Murilla, Qiang Liu, James N. Mutuku, Yao Chen, Arlene S. Bridges, Raymond E. Mdachi, Mohamed A. Ismail, Shelley Ching, David W. Boykin, James Edwin Hall, Richard R. Tidwell, Mary F. Paine, Reto Brun, Michael Zhuo Wang

**Affiliations:** 1 Trypanosomiasis Research Centre, Kenya Agricultural Research Institute (KARI-TRC), Kikuyu, Kenya; 2 University of Basel, Basel, Switzerland; 3 UNC Eshelman School of Pharmacy, The University of North Carolina at Chapel Hill, Chapel Hill, North Carolina, United States of America; 4 Department of Pharmaceutical Chemistry, The University of Kansas, Lawrence, Kansas, United States of America; 5 Department of Pathology and Laboratory Medicine, School of Medicine, The University of North Carolina at Chapel Hill, Chapel Hill, North Carolina, United States of America; 6 Department of Chemistry, Georgia State University, Atlanta, Georgia, United States of America; 7 SVC Associates, Inc., Apex, North Carolina, United States of America; 8 Swiss Tropical and Public Health Institute, Basel, Switzerland; Institute of Tropical Medicine, Belgium

## Abstract

There are no oral drugs for human African trypanosomiasis (HAT, sleeping sickness). A successful oral drug would have the potential to reduce or eliminate the need for patient hospitalization, thus reducing healthcare costs of HAT. The development of oral medications is a key objective of the Consortium for Parasitic Drug Development (CPDD). In this study, we investigated the safety, pharmacokinetics, and efficacy of a new orally administered CPDD diamidine prodrug, 2,5-bis[5-(*N*-methoxyamidino)-2-pyridyl]furan (DB868; CPD-007-10), in the vervet monkey model of first stage HAT. DB868 was well tolerated at a dose up to 30 mg/kg/day for 10 days, a cumulative dose of 300 mg/kg. Mean plasma levels of biomarkers indicative of liver injury (alanine aminotransferase, aspartate aminotransferase) were not significantly altered by drug administration. In addition, no kidney-mediated alterations in creatinine and urea concentrations were detected. Pharmacokinetic analysis of plasma confirmed that DB868 was orally available and was converted to the active compound DB829 in both uninfected and infected monkeys. Treatment of infected monkeys with DB868 began 7 days post-infection. In the infected monkeys, DB829 attained a median C_max_ (dosing regimen) that was 12-fold (3 mg/kg/day for 7 days), 15-fold (10 mg/kg/day for 7 days), and 31-fold (20 mg/kg/day for 5 days) greater than the IC_50_ (14 nmol/L) against *T. b. rhodesiense* STIB900. DB868 cured all infected monkeys, even at the lowest dose tested. In conclusion, oral DB868 cured monkeys with first stage HAT at a cumulative dose 14-fold lower than the maximum tolerated dose and should be considered a lead preclinical candidate in efforts to develop a safe, short course (5–7 days), oral regimen for first stage HAT.

## Author Summary

Development of orally administered medicines for human African trypanosomiasis (HAT) would potentially reduce the need for patient hospitalization, thus lowering healthcare costs. In this study, we investigated the potential of a novel diamidine prodrug, DB868 (CPD-007-10), as an oral treatment for first stage HAT. When administered to uninfected monkeys by oral gavage, DB868 was well tolerated up to a maximum dose of 30 mg/kg/day for 10 days (cumulative dose [CD] = 300 mg/kg). DB868 was absorbed into the systemic circulation and was converted to the active compound DB829 in concentrations that were potentially therapeutic for blood trypanosomes. Subsequently, DB868 was evaluated for efficacy in the first stage vervet monkey model of HAT in which treatment was initiated at 7 days post-infection with *T. b. rhodesiense* KETRI 2537. All infected monkeys were cured, even at the lowest of the three dose regimens tested: 3 mg/kg/day for 7 days (CD = 21 mg/kg), 10 mg/kg/day for 7 days (CD = 70 mg/kg) and 20 mg/kg/day for 5 days (CD = 100 mg/kg). DB868 conversion to DB829 was comparable between uninfected and infected monkeys. In view of its favourable safety and oral efficacy profile, we conclude that DB868 is a suitable candidate for development as a new treatment for first stage HAT.

## Introduction

Human African trypanosomiasis (HAT, sleeping sickness) is caused by two trypanosome species that are transmitted through the bite of blood-sucking tsetse flies (*Glossina spp)*. *Trypanosoma brucei (T. b.) gambiense* is endemic to West and Central Africa, while *T. b. rhodesiense* is endemic to East and Southern Africa [1]. The disease is focal in distribution and is marked by wide temporal and spatial variations in incidence and prevalence [2]–[4]. HAT is characterised by two clinical stages. During the first (early, haemolymphatic) stage, trypanosomes proliferate at the site of the fly bite, travel to local lymph nodes and bloodstream, and progressively invade other tissues [5]. Approximately 3–4 weeks post-infection with *T. b. rhodesiense*, or months to years with *T. b. gambiense*, trypanosomes invade the central nervous system (CNS), initiating the second (late, meningo-encephalitic) stage of HAT [5]. Second stage HAT is marked by neurological and endocrine disorders. If patients are not treated, they lapse into coma and die [6].

HAT chemotherapy is stage specific. Only two drugs have been approved for the treatment of first stage HAT, pentamidine and suramin. Pentamidine, a diamidine first used clinically in 1941, is used to treat first stage *T. b. gambiense* infections. Suramin, a naphthylurea first introduced for clinical use in 1921, is effective against both trypanosome species but is mainly used against first stage *T. b. rhodesiense* HAT [7], [8]. Pentamidine is associated with hypoglycaemia, pain at the injection site, diarrhoea, nausea and vomiting, while suramin is associated with hypersensitivity reactions, albuminuria, haematuria and peripheral neuropathy [7]. In addition, these drugs must be administered *via* intramuscular injection or intravenous infusion in well-equipped hospitals, which are not readily available or accessible in rural areas where HAT typically occurs. To overcome these limitations, two orally active compounds, fexinidazole and the oxaborole SCYX-7158, have recently entered clinical development to treat both stages of the disease [9]. In addition, efforts by the Consortium for Parasitic Drug Development (CPDD) to address these limitations have resulted in the synthesis of a collection of diamidines with promising pharmacologic properties [10], [11].

**Figure 1:**
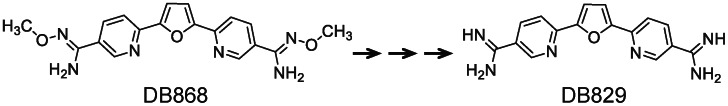
Structures of the prodrug (DB868) and active compound (DB829).

One of the new aza diamidines, 2,5-bis(5-amidino-2-pyridyl)furan (DB829; Figure 1), exhibited an IC_50_ of 14 nmol/L against *T. b. rhodesiense* STIB900 *in vitro* [12]. In addition, it was shown to be 100% curative in both the acute (*T. b. rhodesiense* STIB900) and chronic CNS (*T. b. brucei* GVR35) mouse models of HAT after intraperitoneal administration [12]. However, the dicationic nature of DB829 and other diamidines (*e.g.*, pentamidine and furamidine) contributes to poor permeation through biologic membranes and in turn, poor systemic exposure after oral administration [13]. As such, a prodrug of DB829 was designed, 2,5-bis[5-(*N*-methoxyamidino)-2-pyridyl]furan (DB868; Figure 1), by masking the cationic functionalities of the active compound with methoxy groups [14]. Oral administration of DB868 was 100% curative in both the acute and chronic CNS mouse models of HAT [12]. Based on these desirable properties, DB868 progressed into our vervet monkey model to assess its potential as a new lead compound for oral treatment of first stage HAT. The purpose of this study was to evaluate DB868 metabolism in monkey liver microsomes, safety in uninfected monkeys, pharmacokinetics in both uninfected and infected monkeys, and efficacy in infected monkeys.

## Materials and Methods

### Ethics

This study was conducted in accordance with experimental guidelines and procedures (Ref: C/TR/4/325/116) approved by the Institutional Animal Care and Use Committee (IACUC) at the Kenya Agricultural Research Institute's Trypanosomiasis Research Centre (KARI-TRC). These IACUC regulations conformed to national guidelines provided by the Kenya Veterinary Association.

### Trypanocidal Test and Comparator Drugs

The test drug, 2,5-bis[5-(*N*-methoxyamidino)-2-pyridyl]furan diacetate (DB868; CPD-007-10; Lot #2-JXS-28; Base MW = 366.37; FW = 564.37), was supplied by the University of North Carolina-led CPDD as a yellow powder stored in opaque, water tight bottles. In the laboratory, the drug-containing vials were wrapped in aluminium foil as further protection from light and stored at room temperature. The drug was dissolved fresh daily in distilled de-ionised water (pH 4.5±0.2) at concentrations permitting administration of 2 mL/kg body weight per oral administration. For example, a 15 mg/mL dose solution was prepared for animals receiving a dose of 30 mg/kg and a 10 mg/mL dose solution for a dose of 20 mg/kg. Pentamidine isethionate, supplied by the World Health Organization (WHO), was used as the comparator drug. Pentamidine was dissolved in sterile distilled water and administered intramuscularly at 0.5 mL/kg body weight.

### Experimental Animals

Eighteen vervet monkeys, also known as African green monkeys or *Chlorocebus (Cercopithecus) aethiops*, weighing from 2.0 to 4.5 kg, were acquired from the Institute of Primate Research in Kenya. To ensure animal welfare and ameliorate suffering, upon arrival at KARI-TRC, the monkeys were subjected to standard quarantine procedures, including screening for zoonotic and non-zoonotic diseases/infections and treatment for both endo- and ectoparasites, for a minimum of 90 days prior to study commencement as previously described [15], [16]. They became accustomed to staying in individual squeeze-back stainless steel cages during this time. The monkeys were maintained on a diet of fresh fruits and vegetables (bananas, tomatoes, carrots and green maize) and commercial monkey cubes (Unga Feeds, Nakuru, Kenya) fed twice daily, and were given water *ad libitum*. The commercial monkey cubes were manufactured to have the following nutrient composition: crude protein, 19.4% (w/w); crude fiber, 5.6% (w/w); ether extracts that include fats and lipids, 4.2% (w/w); and nitrogen-free extracts, 66.5% (w/w).

### Metabolism of DB868 in Vervet Monkey Liver Microsomes

DB868 metabolism was studied in male vervet monkey liver microsomes (custom-prepared by XenoTech, LLC, Lenexa, KS, USA) by adapting a previously published method [17]. Briefly, incubation mixtures contained 10 µM DB868, 0.5 mg/mL monkey liver microsomes, and 3.3 mM MgCl_2_ in 100 mM phosphate buffer (pH 7.4). Reactions were initiated by the addition of NADPH (1 mM final concentration). Control incubations were carried out without NADPH, DB868, or liver microsomes. Aliquots (100 µL) of the reaction mixtures were removed at 0, 5, 10, 15, 30, 60 and 120 min and mixed with 100 µL of ice-cold acetonitrile. After centrifugation to pellet precipitated proteins, the supernatants were analyzed by HPLC/UV and fluorescence using the method previously described for pafuramidine (DB289) and furamidine (DB75) [18]. Metabolite identification was performed by comparing retention times to those of synthetic standards for M1 (DB1679), M2 (DB840), M3 (DB1712), and DB829. DB868 and its metabolites were quantified using a calibration curve (0.1–10 µM) generated using synthetic standards.

### Safety and Pharmacokinetics in Uninfected Monkeys

Eight uninfected vervet monkeys, divided into two dose-groups of four monkeys (two females and two males) each, were used. Baseline weight and clinical and haematological data were collected over a 14-day period, after which the monkeys were orally administered DB868 at 10 mg/kg/day (group 1) or 30 mg/kg/day (group 2) for 10 days (Table 1). Care was taken to avoid spillage and to minimise the time between drug preparation and dosing. Drug administration utilized a dose volume of 2 mL/kg body weight. The animals were monitored for indicators of overt toxicity, including changes in feed intake, weight, demeanour, posture and stool composition and consistency. Feed intake was assessed by scoring the proportion of the daily ration consumed by each monkey on a scale of 1 (full ration eaten), ¾, ½, ¼, and 0 (no feed eaten) as previously described [15]. To increase chances of detecting potential drug-related gastrointestinal toxicity, stool samples were collected and examined visually and by faecal occult blood tests conducted according to the modified guaiac method [19]. Post-last drug dose (LDD) monitoring extended to a minimum of 60 days.

**Table 1:** Pharmacokinetics of DB868 and DB829 in uninfected vervet monkeys after the final (10^th^) oral dose of DB868.

Compound	Outcome	Units	Oral DB868							
			10 mg/kg×10 days				30 mg/kg×10 days			
		Monkey ID	643	659	675	677	546	567	668	679
DB868	C_4 h_	nmol/L	50	130	39	250	1600	280	320	330
	C_24 h_	nmol/L	BLQ	BLQ	6	BLQ	38	47	35	66
	AUC_last_	nmol/L•day	NC	NC	NC	NC	530	210	170	190
	AUC_0-∞_	nmol/L•day	NC	NC	NC	NC	540	260	180	200
	t_1/2_	day	NC	NC	NC	NC	1	1	1.3	0.4
DB829	C_4 h_	nmol/L	240	160	180	170	290	300	270	440
	C_24 h_	nmol/L	140	260	120	170	210	280	340	360
	AUC_last_	nmol/L•day	3000	2800	2100	2100	5700	4600	7100	4700
	AUC_0-∞_	nmol/L•day	3700	3700	4100	2400	7000	6200	12100	5600
	t_1/2_	day	30	30	55	18	24	29	47	22

Key: C_4 h_, concentration at 4 h; C_24 h_, concentration at 24 h; AUC_last_, area under the curve from time zero to the last measurable concentration; AUC_0-∞_, area under the curve from time zero to infinite time; t_½_, terminal elimination half-life; BLQ, below limit of quantitation; NC, not calculable.

During pre- and post-dose monitoring, monkeys were anaesthetized by intramuscular injection of ketamine HCl (10–15 mg/kg) to facilitate physical examination, body weight measurements, and sample collection. Blood was collected from the femoral vein *via* inguinal venipuncture as described previously [16] and divided into aliquots: 1 mL blood into EDTA-containing tubes (1.5 mg EDTA/mL blood) for full haemogram determination and 2 mL blood into EDTA-containing tubes for plasma separation. Plasma was separated using a cool spin centrifuge (1500 rpm for 10 min at 4°C). The harvested plasma was divided into aliquots for clinical chemistry determinations (500 µL), prodrug (DB868) and active compound (DB829) concentration measurement (150 µL), and preservation as a stock sample (approximately 250 µL). All plasma aliquots were frozen at −20°C before analysis.

### Efficacy and Pharmacokinetics in Infected Monkeys

The efficacy of DB868 administered orally at 20 mg/kg/day for 5 days was compared to that of pentamidine administered intramuscularly at 4 mg/kg/day for 7 days. To obtain an indication of dose response, DB868 also was evaluated at 10 and 3 mg/kg/day, administered orally for 7 days. The 10 and 3 mg/kg dose regimens were evaluated at a time when a regulator-imposed freeze on the acquisition of non-human primates was in force, hence only 2 animals were available per dose group. In each experiment, a 14-day baseline data collection period was observed, after which the monkeys were infected by intravenous injection of 10^4^ *T. b. rhodesiense* KETRI 2537 trypanosomes diluted from the infected blood of immuno-suppressed donor Swiss white mice [16]. Post-infection monitoring for the development of parasitaemia was initiated at three days post-infection (DPI) while treatment began 7 DPI, subsequent to confirmation of first stage HAT (defined as trypanosomes detectable in blood and not cerebrospinal fluid [CSF], and CSF white cell counts less than 5 cells/mm^3^) [16]. Ear-prick blood samples to determine parasitaemia were collected prior to daily drug administration. Clinical and parasitological cure was evaluated for at least six months as previously described [16]. In our studies with the related prodrug DB844, plasma samples collected out to 28 days post-LDD (6 mg/kg) were insufficient to recover a robust estimate of the elimination half-life of the active compound DB820 [20]; therefore, in the current study, plasma samples were collected for at least 60 days post-LDD for pharmacokinetic analysis. Haematology samples were analysed using an AC^3diff^T Coulter Counter (Miami, FL, USA). Clinical chemistry determinations were performed using a Humalyzer analyser system. Plasma was analyzed for prodrug and active compound concentrations using high performance liquid chromatography-tandem mass spectrometry (HPLC-MS/MS) as described below.

### Handling of Moribund Infected Monkeys

Monkeys that were deemed as treatment failures/relapses or developed severe adverse clinical signs as defined in the protocol (*e.g.*, inability or reluctance to perch, less than ¼ of normal daily feed intake for 2–3 consecutive days) were immediately withdrawn from the study and humanely euthanized for post-mortem examination. These monkeys were euthanized by intravenous administration of 20% (w/v) pentobarbitone sodium solution (150 mg/kg body weight; Euthatal; Rhône-Mérieux, United Kingdom).

### HPLC-MS/MS Quantification of DB868 and DB829 in Monkey Plasma

Monkey plasma samples were processed for quantification of DB868 and DB829 using previously described methods [21], [22] with modifications made to the transition and mass spectrometer parameters. DB868-*d_6_*P (DB868 with deuterated pyridyl rings; 30 nM) and DB829-*d_6_* (DB829 with deuterated pyridyl rings; 30 nM) were used as internal standards and were supplied by the CPDD. Prodrug and active compound were separated on an Aquasil C_18_ HPLC column (50×2.1 mm, 5 µm; Thermo Fisher Scientific, Waltham, MA, USA) and quantified using an Applied Biosystems API 4000 triple quadrupole mass spectrometer equipped with a Turbo V source and electrospray probe (Foster City, CA, USA). The following transitions (in positive ion mode) were used in multiple reaction monitoring scans: 367.1→320.2 (DB868), 373.7→323.2 (DB868-*d_6_*P), 307.1→290.1 (DB829), and 313.2→296.2 (DB829-*d_6_*). Calibration standards and quality controls were prepared in blank monkey plasma to mimic the matrix of the unknown test samples. Analyte concentrations were reported only for those samples that were between the standards and controls that had an accuracy and precision within 100%±20%. If samples were below this range, data are reported as below the limit of quantification. Data below the limit of quantification were not used for the pharmacokinetic analysis.

### Data Analysis

Data were analysed statistically using StatView for Windows Version 5.0.1 (SAS Institute Inc., Cary, NC, USA) as previously published [20]. Repeated measures ANOVA, with Fisher's PLSD post hoc test, was used to test the effects of trypanosomal infection, as well as DB868, on haematological and clinical chemistry parameters in comparison with respective baseline values (α = 0.05). Confidence intervals (95%) were derived to further test the significance of observed findings. The clinical data arising from the efficacy study are presented descriptively since the group sizes were too small for statistical analysis. Pharmacokinetic outcomes were determined with standard non-compartmental methods using Phoenix WinNonlin (version 6.2; Pharsight, Mountain View, CA, USA).

## Results

### Conversion of Prodrug to Active Compound in Monkey Liver Microsomes

**Figure 2:**
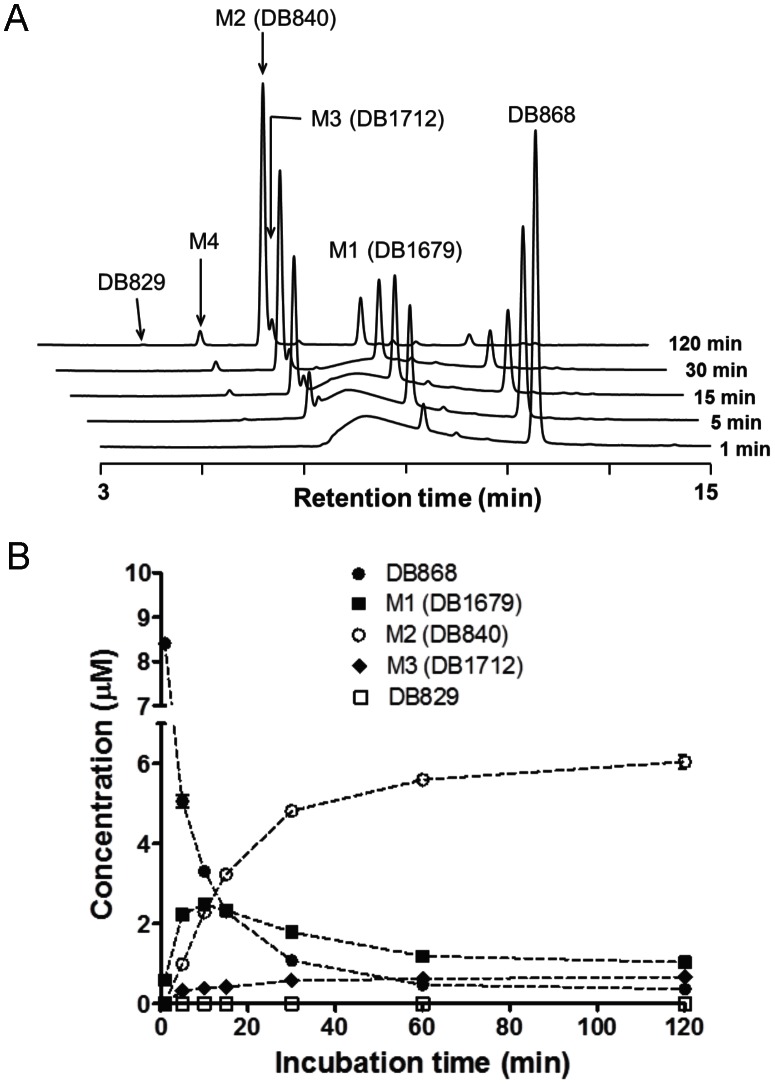
Metabolism of the prodrug DB868 in monkey liver microsomes. HPLC/UV chromatograms (A) and concentration-time profiles (B) of the prodrug DB868, intermediate metabolites, and active compound (DB829) following incubation of DB868 with male vervet monkey liver microsomes were shown. Incubation mixtures (1 mL at pH 7.4) contained 10 µM DB868, 0.5 mg/mL monkey liver microsomes, and 1 mM NADPH. Aliquots were removed at 1, 5, 15, 30 and 120 min, and analyzed for DB868, three intermediate metabolites (M1, M2, M3), and DB829 by HPLC/UV. Metabolite M4 was not quantified due to the lack of a synthetic standard. Symbols and error bars represent means and SDs of triplicate incubations.

The prodrug DB868 was metabolized in male vervet monkey liver microsomes to M1 (DB1679), M2 (DB840), M3 (DB1712), M4, and the active compound DB829 (Figure 2). These metabolites were similar to those observed when DB868 was incubated with human liver microsomes [23]. Detection of these metabolites indicated that, like pafuramidine (DB289) and DB844, DB868 undergoes sequential *O*-demethylation and *N*-dehydroxylation reactions to form the active compound DB829 in monkey liver microsomes. M2 (DB840), a bis-amidoxime metabolite, had the highest concentration at the end of the 120-min incubation. DB829 was at the limit of detection in UV mode; however, formation was confirmed by subsequent parallel fluorescence and mass spectrometric detection (data not shown).

### Overt Toxicity and Haematology in Uninfected Monkeys

**Figure 3:**
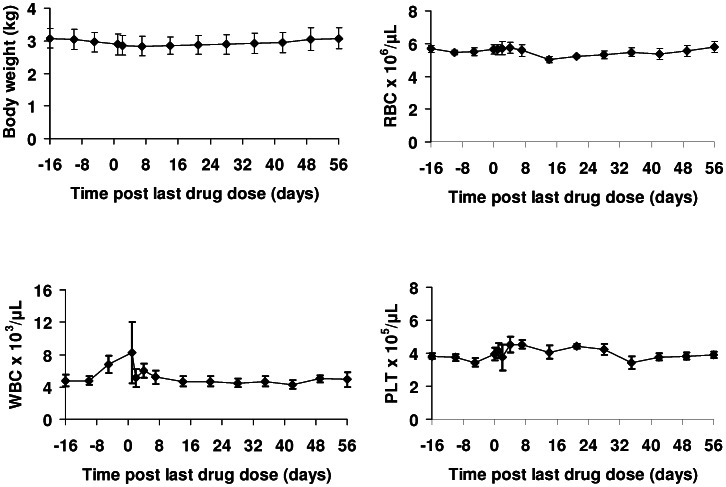
Changes in body weight and haematological parameters in uninfected vervet monkeys administered DB868. The monkeys (n = 4) were administered DB868 orally at 30 mg/kg/day for 10 days, day −9 to day 0 post-last drug dose. Symbols and error bars represent means and SEs, respectively, of body weight, red blood cell count (RBC), white blood cell count (WBC), and platelet count (PLT).

Uninfected monkeys administered DB868 orally at 10 mg/kg/day for 10 days (n = 4) did not exhibit any adverse clinical signs throughout the study. In addition, two of the four monkeys in the 30 mg/kg/day group did not display overt toxicity. The remaining two monkeys exhibited mild signs of toxicity, including excess mucous in the stool (monkey 567) and transient inappetance 1–2 days post-LDD (monkeys 567 and 546). In general, stool texture and consistency was unchanged and faecal occult blood tests revealed nothing significant in any study subject, suggesting that no notable gastrointestinal toxicity occurred. The mean body weight of monkeys in the 30 mg/kg group exhibited minimal variation (Figure 3), with a maximum decline of 6.5% from baseline (3.1 kg±0.3). A maximum decline of 6.2% from baseline (3.2 kg±0.5) was observed in the 10 mg/kg group (data not shown). Overall, the two oral DB868 dose regimens were well tolerated.

Haematological parameters of the two treatment groups did not vary significantly from baseline throughout the study. For the 30 mg/kg group, the baseline mean red blood cell (RBC) and platelet counts (± SE) were 5.5 (±0.1)×10^6^ and 3.7 (±0.2)×10^5^ cells/µL of blood, respectively, and showed little change throughout the study (*p* = 0.10 and 0.06, respectively; Figure 3). Similarly, no significant variations were seen in the RBC or platelet counts for the 10 mg/kg group (data not shown). The mean white blood cell (WBC) count exhibited a minor transient increase post-LDD (1.5-fold over the baseline count (± SE) of 4.8 (±0.5)×10^3^ cells/µL of blood; *p* = 0.05; Figure 3), which returned to baseline after 24 h. A comparable trend was seen in the WBC count of the 10 mg/kg group (data not shown), as well as in a separate monkey that was not administered drug or vehicle but had blood sampled at the same time as monkeys in the current experiment, suggesting that the change in the WBC count was not drug-related.

### Clinical Chemistry

**Figure 4:**
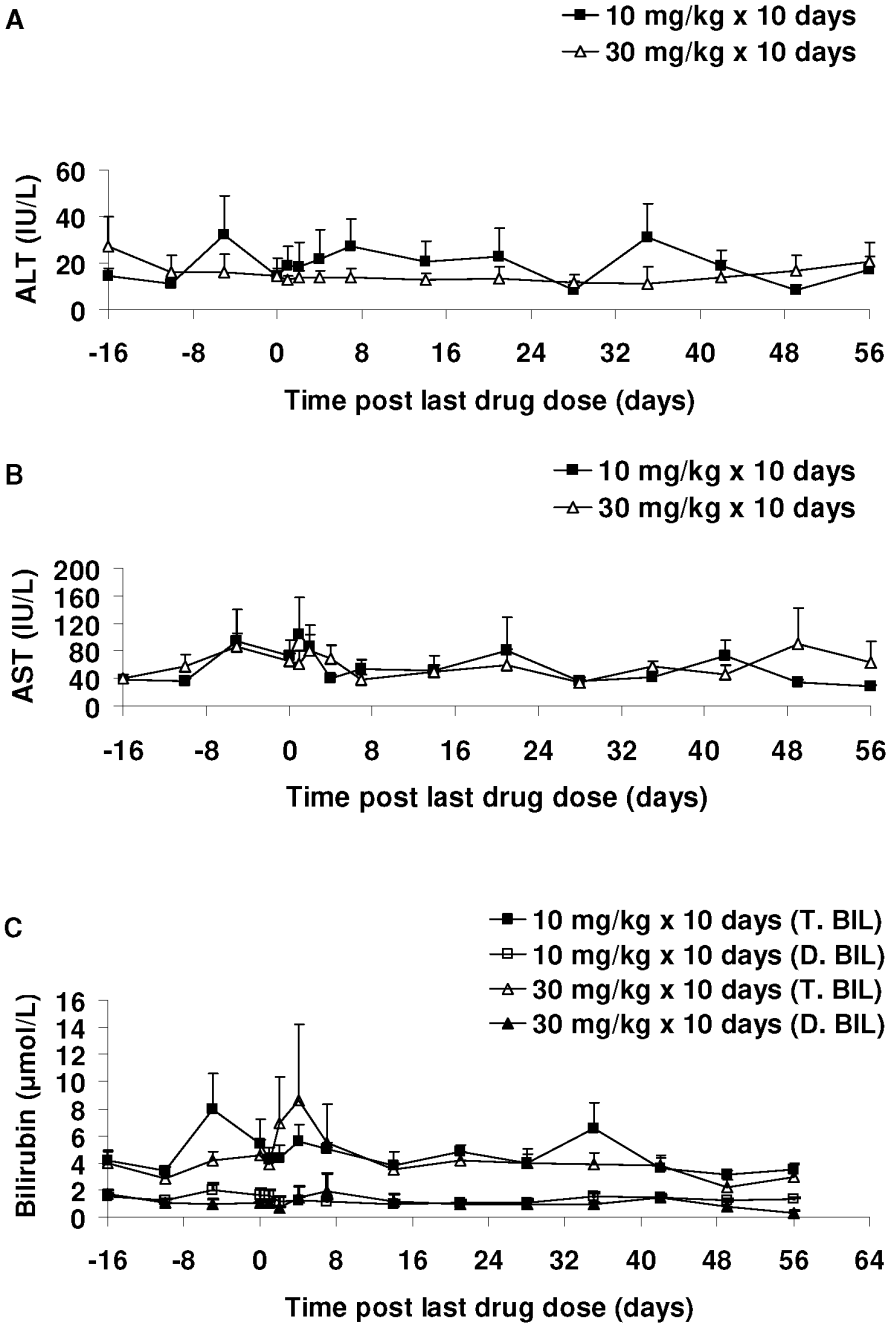
Changes in plasma biomarkers of liver injury in uninfected vervet monkeys administered DB868. DB868 was administered orally at 10 mg/kg/day (n = 4) or 30 mg/kg/day (n = 4) for 10 days, day −9 to day 0 post-last drug dose. Symbols and error bars represent means and SEs, respectively, of (**A**) alanine aminotransferase (ALT), (**B**) aspartate aminotransferase (AST), and (**C**) total and direct bilirubin (T. BIL and D. BIL, respectively) levels.

Plasma biomarkers of liver injury, alanine aminotransferase (ALT), aspartate aminotransferase (AST) and total and direct bilirubin, were monitored in uninfected monkeys prior to (baseline), during, and following completion of the 10-day DB868 dosing regimens. At the two baseline time points (−16 and −10 days post-LDD), mean (± SE) ALT levels were 14.6 (±3.1) and 11.1 (±2.3) IU/L, respectively, for the 10 mg/kg group and 27.3 (±13.0) and 16.0 (±7.2) IU/L for the 30 mg/kg group. During and following the completion of dosing, mean ALT levels varied considerably in the 10 mg/kg group (Figure 4A). The highest post-treatment mean ALT values observed were 2.8-fold (31.3/11.1) greater than the second baseline sample for the 10 mg/kg group and 1.3-fold (20.5/16.0) for the 30 mg/kg group. Neither variation was statistically significant (*p* = 0.71, 10 mg/kg group; *p* = 0.37, 30 mg/kg group). Mean AST and total and direct bilirubin levels also exhibited variability prior to, during, and following dosing (Figure 4, B and C) but overall, were not statistically different from baseline values (*p*>0.05).

**Figure 5:**
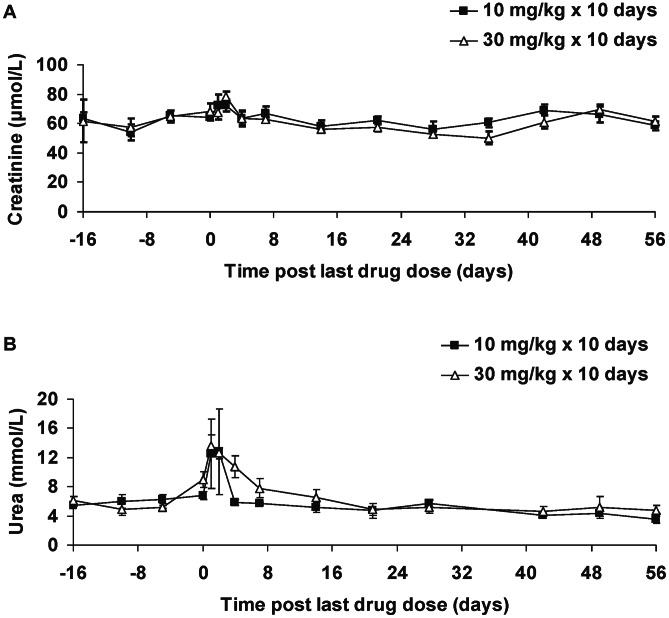
Changes in plasma biomarkers of kidney injury in uninfected vervet monkeys administered DB868. DB868 was administered orally at 10 mg/kg/day (n = 4) or 30 mg/kg/day (n = 4) for 10 days, day −9 to day 0 post-last drug dose. Symbols and error bars represent means and SEs, respectively, of (**A**) creatinine and (**B**) urea.

Two biomarkers of kidney injury, creatinine and urea, were also evaluated in plasma samples. Mean (± SE) creatinine concentrations at the two baseline time points were 63.6 (±4.0) and 54.1 (±5.5) µmol/L, respectively, for the 10 mg/kg group and 61.5 (±14.5) and 57.3 (±6.0) µmol/L for the 30 mg/kg group. Post-dosing variations were minimal (Figure 5A) and not statistically significant (*p*>0.05). However, mean urea levels exhibited a transient increase following the completion of each dosing regimen (Figure 5B; Figure S1). Plasma urea peaked 1–2 days post-LDD and was 2.1- and 2.7-fold greater than baseline for the 10 mg/kg and 30 mg/kg groups, respectively (both *p*<0.05). To determine whether increases in plasma urea levels were due to kidney dysfunction, the blood urea nitrogen (BUN)∶creatinine ratio was calculated for the 1–2 days post-LDD period. The peak mean urea concentration for the 10 mg/kg group was 12.8 mmol/L, which is equivalent to 35.9 mg/dL of BUN. The mean creatinine concentration was 72.3 µmol/L (equivalent to 0.8 mg/dL). Therefore, the BUN∶creatinine ratio was 45∶1 (35.9∶0.8). The BUN∶creatinine ratio for the 30 mg/kg group was higher at 48∶1.

### Pharmacokinetics in Uninfected Monkeys

**Figure 6:**
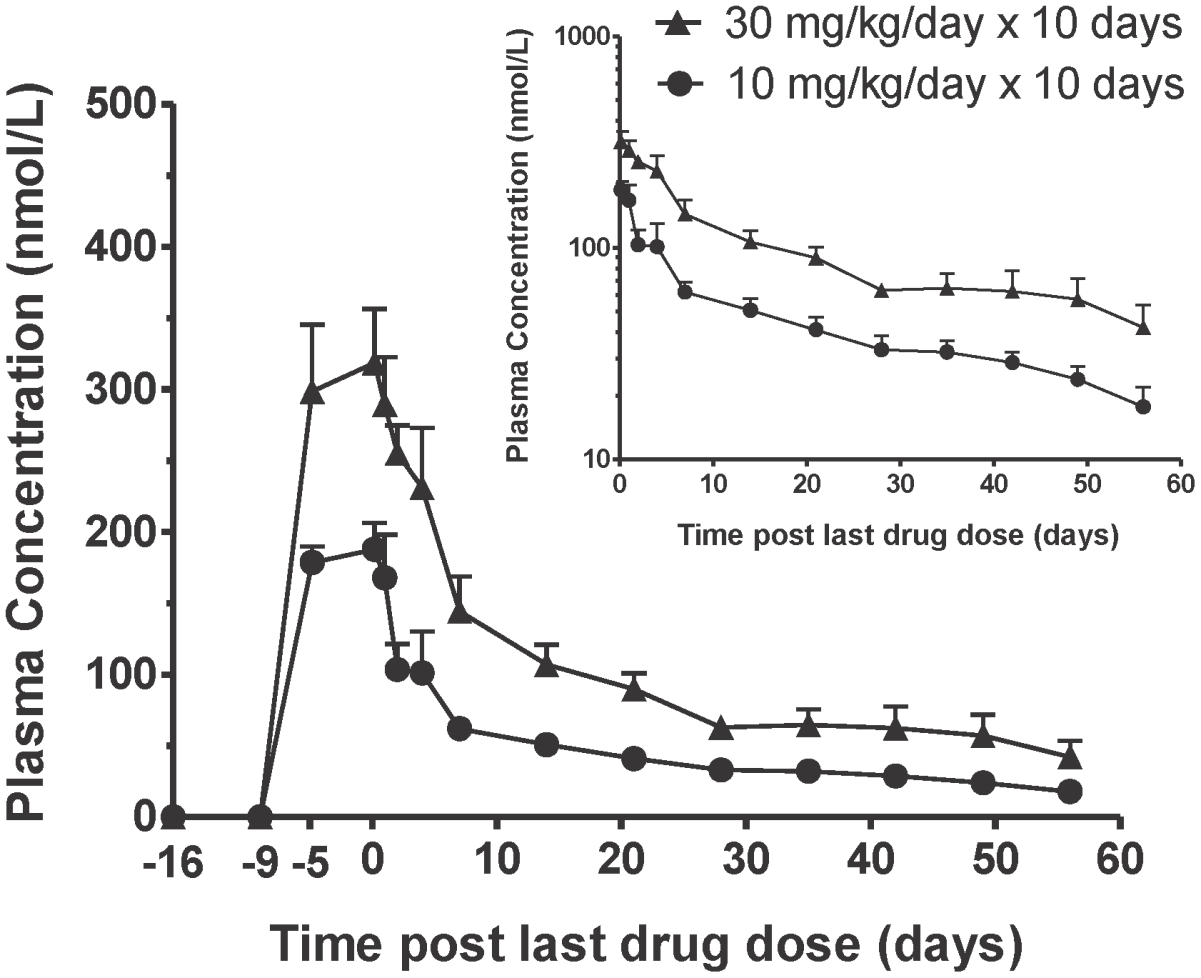
Plasma concentration-time profiles of DB829 following administration of the prodrug DB868 to uninfected vervet monkeys. DB868 was administered orally at 10 mg/kg/day (n = 4) or 30 mg/kg/day (n = 4) for 10 days, day −9 to day 0 post-last drug dose. Symbols and error bars represent geometric means and SEs of the active compound DB829 concentrations, respectively. The inset graph shows the plasma concentration-time profiles starting at day 0 post-last drug dose on a logarithmic scale.

Following oral administration of the prodrug DB868 to uninfected monkeys, DB868 was detected in plasma at 4 h post-LDD (Figure 6; Table 1). DB868 concentrations declined to below the limit of detection (BLD) within 1–2 days post-LDD for the 30 mg/kg group and in less than 1 day post-LDD for the 10 mg/kg group (data not shown). Accurate recovery of pharmacokinetic outcomes for DB868 was precluded for the 10 mg/kg dose group (Table 1). Greater inter-individual variability was observed for the 4 h post-LDD concentration (C_4 h_) of DB868 than for DB829 (Table 1). The geometric mean DB868 C_4 h_ for the 30 mg/kg group (466 nmol/L) was 5.2-fold greater than that for the 10 mg/kg group (89 nmol/L). The geometric mean DB829 C_4 h_ for the 30 mg/kg group (320 nmol/L) was 1.6-fold greater than that for the 10 mg/kg group (185 nmol/L). The geometric mean AUC_last_ and AUC_0-∞_ for DB829 in the 30 mg/kg group were 2.2-fold greater than that in the 10 mg/kg group. The geometric mean terminal elimination half-life for DB829 was comparable between the two dose groups (29 and 31 days, respectively).

### Disease Progression and Efficacy

**Figure 7:**
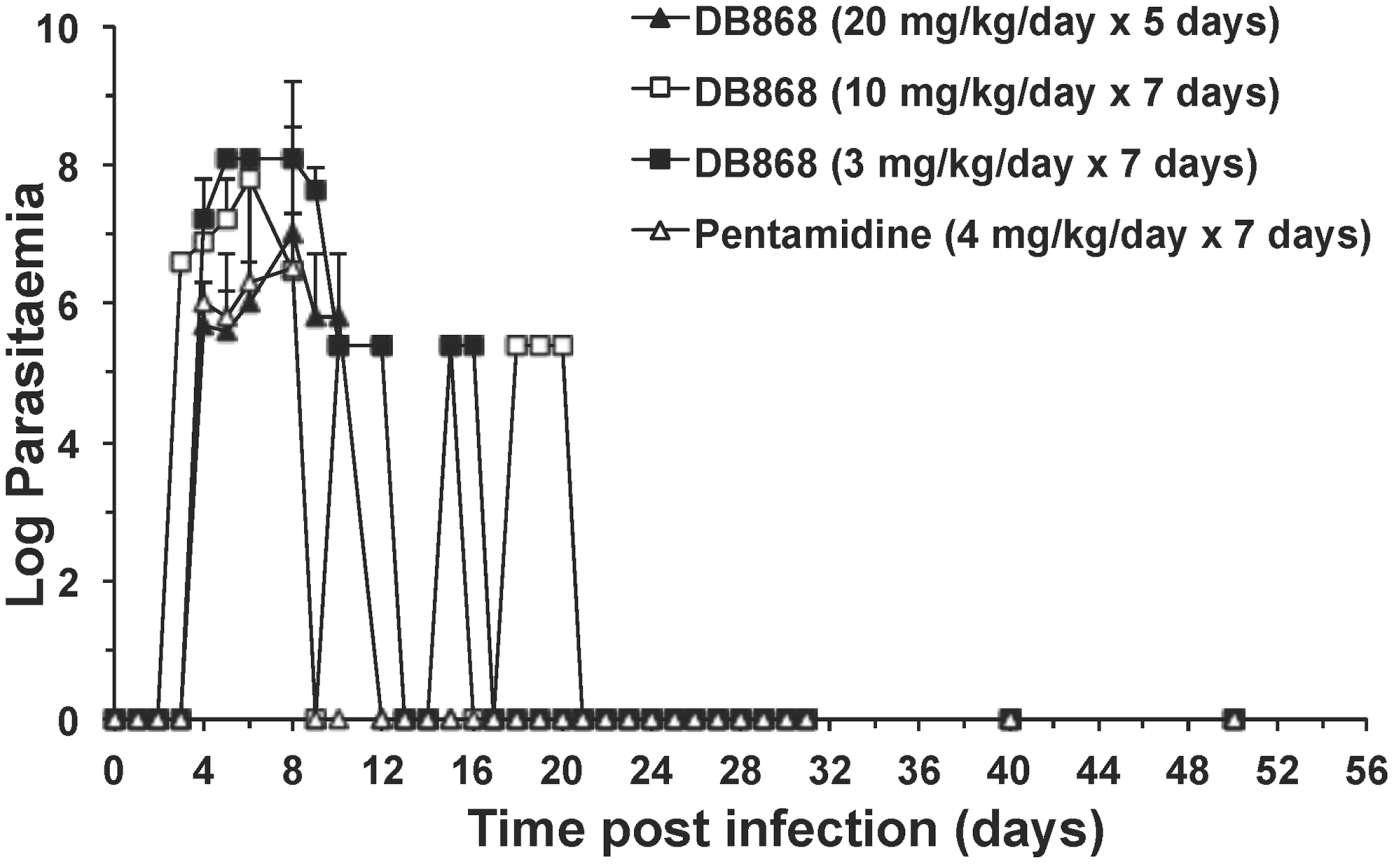
Changes in mean parasitaemia values of vervet monkeys treated with DB868. Starting at 7 days post-infection with *T. b. rhodesiense* KETRI 2537, monkeys confirmed to have first stage HAT were treated with either DB868 orally or pentamidine intramuscularly. DB868 at 20 mg/kg/day for 5 days (n = 2); DB868 at 10 mg/kg/day for 7 days (n = 2); DB868 at 3 mg/kg/day for 7 days (n = 2); pentamidine at 4 mg/kg/day for 7 days (n = 3). Symbols and error bars represent means and interindividual differences (range), respectively.

Following inoculation, the median (range) prepatent period of *T. b. rhodesiense* infection in the monkeys was 4.5 (3–6) days (Table 2; Figure 7). The bloodstream form of *T. b. rhodesiense* KETRI 2537 trypanosomes multiplied rapidly, reaching a peak mean count of 1.1×10^7^ trypanosomes/mL blood; in some monkeys, the count peaked as high as 1.3×10^8^ trypanosomes/mL (antilog 8.1; Table 2). Classical signs of *T. b. rhodesiense* infection were observed, including rough hair coat, dullness, marked loss of appetite, and marginal declines in body weight (4% of pre-infection weight) and RBC count (7% of pre-infection value). Rectal body temperature increased from a pre-infection mean (± SE) of 38.3 (±0.2)°C to a high of 38.7 (±0.2)°C at 7 DPI; however, the increase was not statistically significant (*p* = 0.06). Trypanosomes were not detected, nor were white cell counts elevated in the CSF (data not shown), confirming that the monkeys were in the first stage of disease when treatment was initiated at 7 DPI. The prodrug DB868 was administered orally to three groups of monkeys: 20 mg/kg/day for 5 days (n = 3), 10 mg/kg/day for 7 days (n = 2), or 3 mg/kg/day for 7 days (n = 2). A fourth group of monkeys (n = 3) was treated intramuscularly with the comparator drug, pentamidine, at 4 mg/kg/day for 7 days (Table 2). Both oral DB868 and intramuscular pentamidine demonstrated efficacy against first stage infection as discussed below. Trypanosome-associated waves of parasitaemaia were not observed (Figure 7), likely because all infections were treated during the first wave of parasitaemia.

**Table 2:** Efficacy of oral DB868 and intramuscular pentamidine against first stage *T. b. rhodesiense* infection in vervet monkeys.

Parameter/Outcome	Oral DB868							Intramuscular Pentamidine		
	20 mg/kg×5 days			10 mg/kg×7 days		3 mg/kg×7 days		4 mg/kg×7 days		
Monkey ID	585	658	686	675	691	638	643	651	541	672
Prepatent period (days post-infection)	5	5	4	5	3	4	4	4	5	6
Peak parasitaemia (Log_10_ P)	7.2	6.9	6.0	7.8	7.8	8.1	8.1	5.4	7.8	5.4
Trypanosomes blood/CSF at EoT	Neg	Neg	Neg	Neg	Neg	Neg	Neg	Neg	Neg	Pos
Provisional efficacy at 100 days post-treatment	Cured	Cured	WD	Cured	Cured	Cured	Cured	Cured	Cured	WD
Duration of post-treatment monitoring	145	620	4	114	525	525	525	620	620	2
Final efficacy assesment	WD	Cured	WD	WD	Cured	Cured	Cured	Cured	Cured	Not cured

Key: P = parasitaemia; EoT = end of treatment; Neg = negative; Pos = positive; WD = withdrawn.

#### DB868 at 20 mg/kg/day×5 Days

Three monkeys, 585, 658 and 686, were treated orally with DB868 at 20 mg/kg/day for 5 days. In all monkeys, trypanosomes were undetectable in blood by direct microscopy or the haematocrit centrifugation technique [24] by the 4^th^ day of drug administration. The monkeys remained trypanosome-free in body fluids (blood and CSF) for the remaining monitoring period (Table 2). Monkeys 686 and 585 were withdrawn from the study and humanely euthanized 4 and 145 days post-LDD, respectively (Table 2), due to the continued deterioration of their health. Trypanosome recrudescence had not occurred in either monkey. Upon post-mortem examination, monkey 686 had peritoneal abscesses while monkey 585 had pneumonia, suggesting that their decline in health was unrelated to drug therapy. The remaining monkey (monkey 658) was monitored over 525 days post-LDD without any parasitological or clinical evidence of relapse and was declared cured.

#### DB868 at 10 mg/kg/day×7 Days

Upon treatment with 10 mg/kg/day DB868 orally for 7 days, monkey 691 was parasitologically negative on the 6^th^ day of treatment while monkey 675 became consistently negative 7 days post-LDD. The two monkeys remained negative throughout the remaining post-treatment monitoring period (Table 2). Monkey 675, however, was only monitored up to 114 days post-LDD, at which time it was withdrawn from the study and euthanized due to clinical deterioration. Post-mortem examination indicated pneumonia to be the cause of declining health. Monkey 691 was monitored over 525 days post-LDD without a relapse and was declared cured.

#### DB868 at 3 mg/kg/day×7 Days

Two monkeys, 638 and 643, were treated orally with DB868 at 3 mg/kg/day for 7 days. Monkey 638 was parasitologically negative on the 6^th^ day of dosing, while monkey 643 was 3 days post-LDD. Both monkeys remained trypanosome-free throughout the extended monitoring period of greater than 500 days and were declared cured (Table 2).

#### Pentamidine at 4 mg/kg/day×7 Days

Three monkeys, 541, 651 and 672, were administered pentamidine intramuscularly at 4 mg/kg/day for 7 days. Trypanosomes were not detected in monkeys 541 and 651 immediately prior to the third dose. However, trypanosomes were observed intermittently in monkey 672, including 3 days post-LDD when its clinical condition deteriorated, necessitating euthanasia. Monkeys 541 and 651 were monitored over 600 days post-LDD without any evidence of relapse and were declared cured (Table 2).

### Pharmacokinetics of DB868 and DB829 in Infected Monkeys

**Figure 8:**
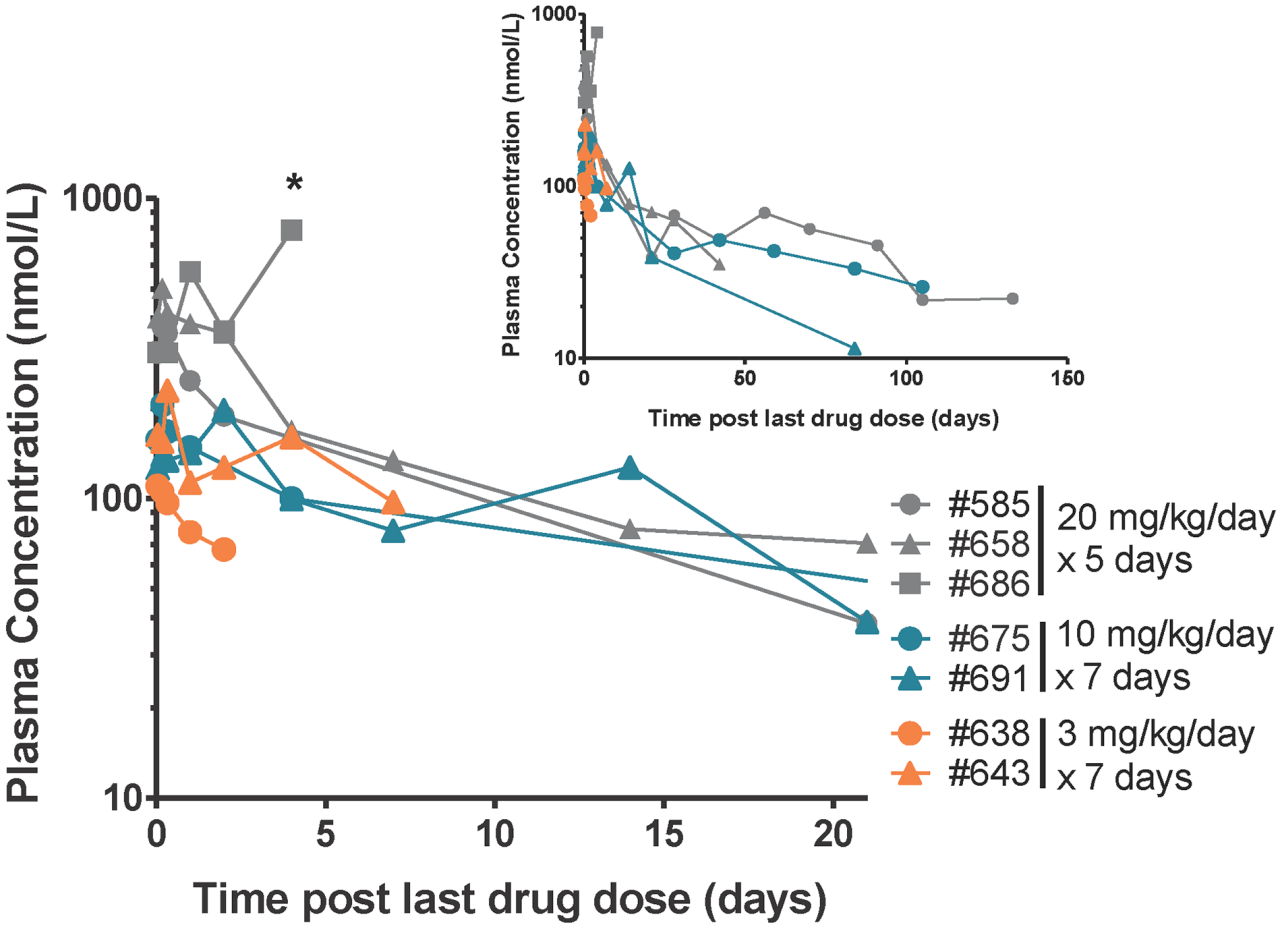
Plasma concentration-time profiles of DB829 following administration of the prodrug DB868 to infected vervet monkeys. The monkeys confirmed to have first stage HAT were administered DB868 orally, beginning at 7 days post-infection, at 20 mg/kg/day for 5 days, 10 mg/kg/day for 7 days, or 3 mg/kg/day for 7 days. The inset graph shows the extended DB829 profiles up to 150 days post-last DB868 dose. * denotes the time (4 days post-last drug dose) that monkey 686 was euthanized due to clinical morbidity (peritoneal abscesses).

The prodrug DB868 was detected in the plasma of all monkeys with first stage HAT, regardless of the dosing regimen, at 0.04 days (1 h) post-LDD (data not shown). The T_max_ varied between individuals, with the majority (4/6 monkeys) occurring at 0.04 days (1 h) post-LDD. DB868 concentrations declined rapidly. Only one monkey (monkey 675; 10 mg/kg/day for 7 days) had detectable levels at 8 h post-LDD, precluding accurate recovery of pharmacokinetic outcomes for DB868. The median C_max_ for DB829 for the 20 and 10 mg/kg groups (435 and 205 nmol/L, respectively) were 2.6- and 1.2-fold higher, respectively, than that for the 3 mg/kg group (170 nmol/L) (Figure 8; Table 3). The median T_max_ for the 20 and 3 mg/kg groups were similar (4 h), whereas that for the 10 mg/kg group was longer (1 day). The median T_max_ for DB829 in each dose group was longer than that for DB868. The mean AUC_last_ for DB829 in the 20 and 10 mg/kg groups was 10- and 7-fold greater, respectively, than that in the 3 mg/kg group (Table 3). Accurate AUC_0-∞_ and terminal elimination half-life were only recoverable for the 20 mg/kg group and one monkey in the 10 mg/kg group, precluding between-dose comparisons. The median AUC_0-∞_ and terminal elimination half-life for the 20 mg/kg group were twice those of the one (monkey 691) in the 10 mg/kg group (Table 3).

**Table 3:** Pharmacokinetics of DB829 in vervet monkeys with first stage HAT after the final oral dose of DB868.

Outcome	Units	Oral DB868					
		20 mg/kg×5 days		10 mg/kg×7 days		3 mg/kg×7 days	
	Monkey ID	585	658	675	691	638	643
C_max_	nmol/L	370	500	210	200	110	230
T_max_	day	0.17	0.17	0.17	2.0	0.04	0.33
AUC_last_ †	nmol/L•day	7300	4100	4000	3500	160	950
AUC_0-∞_	nmol/L•day	10000	5300	NC	3900	NC	NC
t_1/2_	day	85	24	NC	25	NC	NC

Key: C_max_, maximum concentration; T_max_, time to reach maximum concentration; AUC_last_, area under the curve from time zero to the last measurable concentration; AUC_0-∞_, area under the curve from time zero to infinite time; t_½_, terminal elimination half-life; NC, not calculable due to >30% extrapolation of the AUC_0-∞_;

† = last measurable concentration varied between monkeys (2–133 days).

## Discussion

Consistent with observations involving human liver microsomes [23], vervet monkey liver microsomes metabolized the prodrug DB868 to four intermediate metabolites and the active compound DB829. Similar metabolic pathways have been reported for the related prodrugs pafuramidine and DB844 in rat, monkey and human liver microsomes [20], [25]–[27], demonstrating that these alkoxy-type diamidine prodrugs [28] are converted to active compounds in different animal species. The low DB829 concentrations in the microsomal samples were not unexpected, as similar results were observed with the active compound generated from the related prodrug pafuramidine [17]. The final metabolic steps in the formation of DB829, the *N*-hydroxylation of M2 and subsequently M4, are analogous to those in the conversion of pafuramidine to furamidine. During microsomal pafuramidine metabolism, these steps are catalyzed by cytochrome b_5_/b_5_ reductase [29]. This enzyme is also abundant in mitochondria and Golgi [29], [30], explaining why DB868 is more efficiently converted to DB829 in intact hepatocytes compared to the isolated microsomal system [27], [31]. Collectively, these results provided justification for *in vivo* testing of the prodrug DB868 in uninfected and infected vervet monkeys.

No significant overt toxicity was seen with up to 30 mg/kg/day DB868 orally for 10 days (cumulative dose [CD] = 300 mg/kg) in the vervet monkey safety study, suggesting that this dose was below the maximum tolerated dose, but slightly above the no observed adverse effect level (NOAEL). Pharmacokinetic analysis of plasma from the uninfected monkeys showed that the geometric mean C_4 h_ of the active compound DB829 in the 30 and 10 mg/kg groups were 23- and 13-fold greater than the IC_50_ (14 nmol/L) against *T. b. rhodesiense* STIB900, respectively. These results confirmed that DB868/DB829 are available systemically following oral administration, similar to pafuramidine/furamidine and DB844/DB820 [20], [32], prompting further evaluation in the monkey model of first stage HAT.

Based on the above observations, DB868 efficacy was evaluated in vervet monkeys with first stage HAT using doses below 30 mg/kg/day in order to minimise the risk of unfavourable clinical outcomes. Dosing durations of 5–7 days were chosen based on the hypothesis that first stage disease can be cured using short treatment durations. DB868, administered orally, cured all monkeys of their experimentally introduced *T. b. rhodesiense* infection (Table 2). Post-treatment monitoring must be at least 180 days in order to declare a cure in the monkey model of first stage HAT [16]. All three DB868 dosing regimens, including the lowest evaluated (3 mg/kg/day for 7 days; CD = 21 mg/kg), effectively cleared the monkeys of their considerably high parasitaemia, which in some cases was as high as 10^8^ trypanosomes/mL of blood. Elimination of the pathogens allowed the monkeys to return to their clinical and haematological baselines within one month post-LDD (data not shown), similar to what was observed in pafuramidine and DB844 efficacy studies conducted in this monkey model [16], [20]. These results highlight the ability of diamidines to eliminate injurious trypanosomes, allowing the body to repair/heal itself. Furthermore, oral DB868 appears to be superior to oral pafuramidine in this first stage HAT monkey model, as a higher dose of pafuramidine than DB868 was required to achieve complete cure (10 mg/kg for 5 days *vs.* 3 mg/kg for 7 days, respectively) [16]. The longer dose regimen for DB868 compared to pafuramidine is consistent with the *in vitro* observation that DB829 required longer exposure periods than furamidine to kill *T. b. brucei* s427 trypanosomes [33]. For example, a 24-h exposure to DB829 (2.7 µM) was required, whereas a 1-h exposure to furamidine (3.2 µM) was required to kill these trypanosomes in culture.

Based on a mg dose basis, DB868 has a larger therapeutic window than pafuramidine in vervet monkeys. No notable drug-induced overt toxicity was observed in either uninfected or infected monkeys administered DB868, except for mild excess mucous in the stool (n = 1) and transient inappetance (n = 2) in the group receiving 30 mg/kg/day for 10 days. In comparison, similar mild adverse events were observed when pafuramidine was administered at 10 mg/kg/day for 10 days to vervet monkeys (unpublished data; JK Thuita). DB868, at all doses tested, did not cause significant elevations in plasma biomarkers of liver (ALT, AST, total and direct bilirubin; Figure 4) and kidney (creatinine; Figure 5) injury. These results contrasted with those of pafuramidine, which caused transient liver injury during an extended phase I clinical trial in humans [11]. In addition, only a slight increase in ALT (less than 2-fold) was observed in female Sprague-Dawley rats administered DB868 orally (25 mg/kg/day for 3 weeks) compared to untreated rats, whereas an 18-fold increase was observed in rats administered pafuramidine (12 mg/kg/day for 4 weeks) [34]. In the current study, plasma urea concentrations, and therefore BUN levels, were transiently increased (2–3-fold; Figure 5B) shortly (1–2 days) after the last drug dose. However, the BUN∶creatinine ratio was above the critical 20∶1 ratio, suggesting that the elevations were likely due to pre-renal causes such as dehydration. Direct comparison of plasma liver and kidney injury biomarkers between DB868 and pafuramidine in vervet monkeys are not possible due to insufficient data on pafuramidine. Nevertheless, the safety profile of DB868 is improved over that of the prodrug DB844, which caused significant liver injury when administered to monkeys at doses above 10 mg/kg/day [20], necessitating withdrawal of DB844 from further development.

As discussed above, our study has demonstrated that oral DB868 has excellent efficacy and an improved therapeutic window in the first stage HAT monkey model, making it a promising lead candidate for further preclinical development. However, based on the previous lessons learned from the development of pafuramidine [11], several issues warrant mention. First, the kidney safety liability of DB868 needs to be further examined using more predictive models and biomarkers. Pafuramidine development was terminated due to an unexpected severe kidney injury that occurred in five patients (∼6%), a liability not predicted by traditional preclinical safety testing in rodents [11]. Recently, Harrill *et al*. [21] showed, using a mouse diversity panel comprised of 34 genetically diverse inbred mouse strains, marked elevations of urinary kidney injury molecule-1 (KIM-1) in sensitive mouse strains following oral administration of pafuramidine, while classical kidney injury biomarkers, BUN and serum creatinine, remained unchanged. Hence, it may be prudent to screen DB868 for kidney injury liability using the sensitive mouse strains therein identified and KIM-1. Encouraging results from Sprague Dawley rats administered pafuramidine or DB868 orally (12 mg/kg/day×28 days) showed that DB868 had no effect on KIM-1 during the entire 4-month observation period (28 days of drug administration and 92 days of recovery period), whereas pafuramidine caused a 13-fold increase in KIM-1 one week post-LDD [34].

Second, treatment regimens should be optimized with the pharmacokinetics taken into consideration. The active compound DB829 was readily detected in the plasma following oral administration of the prodrug DB868 (Figures 6 and 8). Afterwards, DB829 was slowly eliminated from the blood with a terminal elimination half-life ranging from days to nearly three months depending on the dosing regimen (Tables 1 and 3). This is similar to suramin [7], the only other first stage HAT drug besides pentamidine. Plasma concentrations of DB829 remained >100 nmol/L for long periods following the last DB868 dose, in some monkeys up to 7 days post-LDD. This finding was comparable to that reported for DB844 [20] and provides additional evidence that 1) prolonged treatment durations may not be necessary, especially for first stage HAT, and 2) daily dosing of DB868 and other diamidine prodrugs may not be necessary. However, since trypanosomes are tissue invasive, a follow-up pharmacokinetic study is needed to determine if plasma active drug concentrations are predictive of tissue concentrations.

Third, combined treatment of DB868 with a fast-acting trypanocide may accelerate recovery, improve efficacy and clinical outcomes, and prevent resistance. The time to clearance of trypanosomes from peripheral blood was shorter in monkeys treated with pentamidine intramuscularly (2 days after the 1^st^ 4 mg/kg dose) than with oral DB868 (2–14 days after the 1^st^ dose depending on the dose; Figure 7). It took longer for the lower DB868 dose regimen groups (3 and 10 mg/kg) to clear parasites from the blood than the 30 mg/kg group (6–14 days *vs.* 2–5 days after the 1^st^ drug dose; Figure 7). The difference in parasite clearance between pentamidine and DB868 (or the active compound DB829) is consistent with observations in mouse models of HAT (Wenzler *et al.*, *Antimicrob Agents Chemother*. under review). However, the slower parasite clearance by DB868 did not seem to compromise efficacy in the monkey model. Nevertheless, combining oral DB868 with another fast-acting trypanocidal agent, such as the oral drugs currently in clinical trials, may offer fast elimination of parasitaemia, the ease of oral pills, and a low probability of developing resistance.

In conclusion, oral DB868 demonstrated improved efficacy and safety profiles in the vervet monkey model of first stage HAT, in comparison to the previous clinical candidate pafuramidine. As such, DB868 should be considered a preclinical candidate for oral treatment of first stage HAT, supplementing the current drug development pipeline.

## Supporting Information

Figure S1 **Individual plasma urea concentration-time profiles of uninfected vervet monkeys administered DB868.** DB868 was administered orally at 10 mg/kg/day (643, 675, 659, 677; blue symbols) or 30 mg/kg/day (567, 679, 546, 668; red symbols) for 10 days, day −9 to day 0 post-last drug dose.
(TIF)

Figure S2 **Individual plasma concentration-time profiles of DB829 following administration of DB868 to uninfected vervet monkeys.** DB868 was administered orally at 10 mg/kg/day (643, 675, 659, 677; blue symbols) or 30 mg/kg/day (567, 679, 546, 668; red symbols) for 10 days day −9 to day 0 post-last drug dose.
(TIF)
