# Supplementary figures and images for: Safety, Pharmacokinetic, and Efficacy Studies of Oral DB868 in a First Stage Vervet Monkey Model of Human African Trypanosomiasis
Source: PLoS Negl Trop Dis. 2013 Jun 6;7(6):e2230. doi: 10.1371/journal.pntd.0002230 (PMC3674995; doi:10.1371/journal.pntd.0002230)

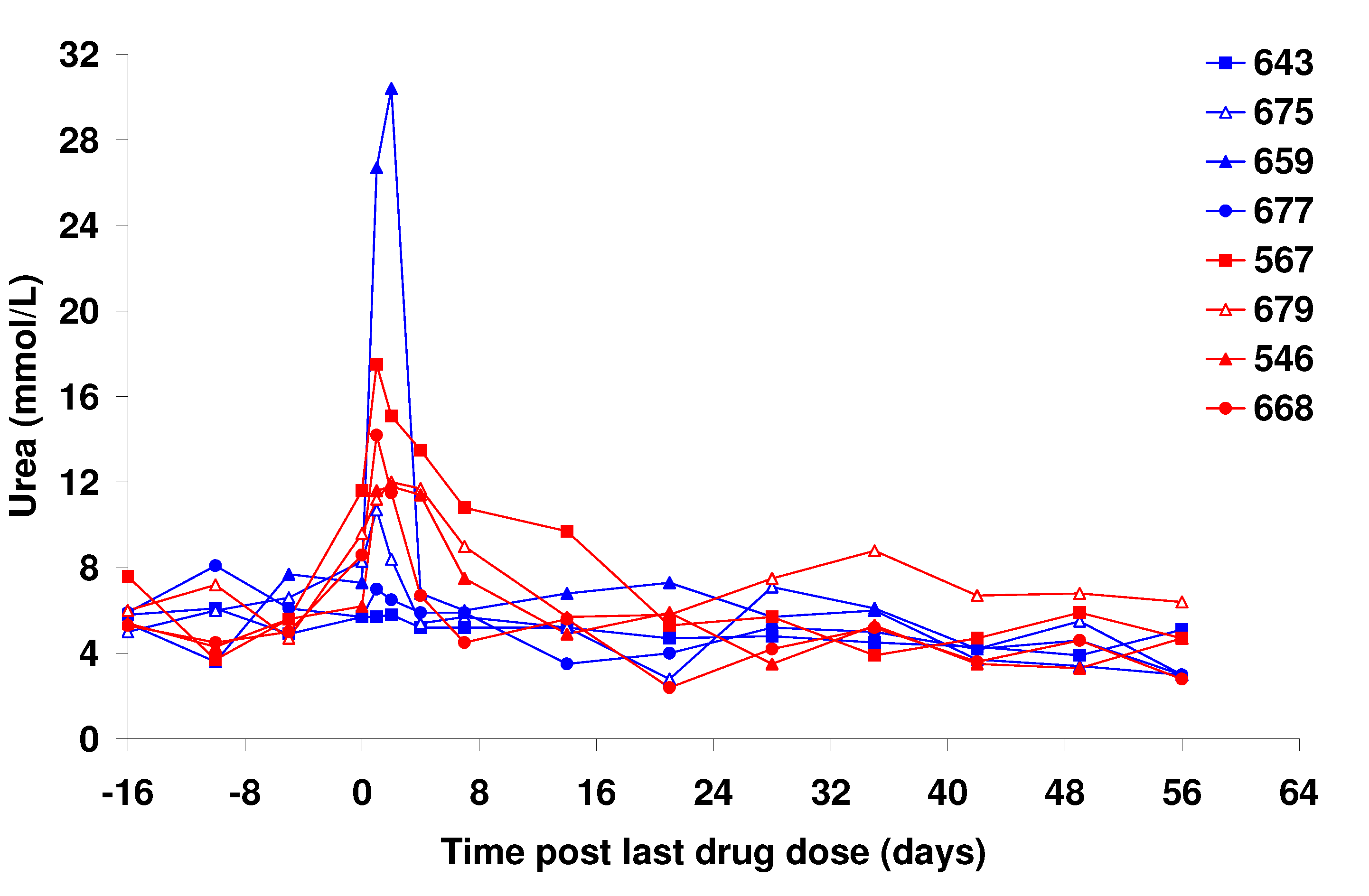

Supplement: Figure S1 — Individual plasma urea concentration-time profiles of uninfected vervet monkeys administered DB868. DB868 was administered orally at 10 mg/kg/day (643, 675, 659, 677; blue symbols) or 30 mg/kg/day (567, 679, 546, 668; red symbols) for 10 days, day −9 to day 0 post-last drug dose. (TIF) [file pntd.0002230.s001.tif]

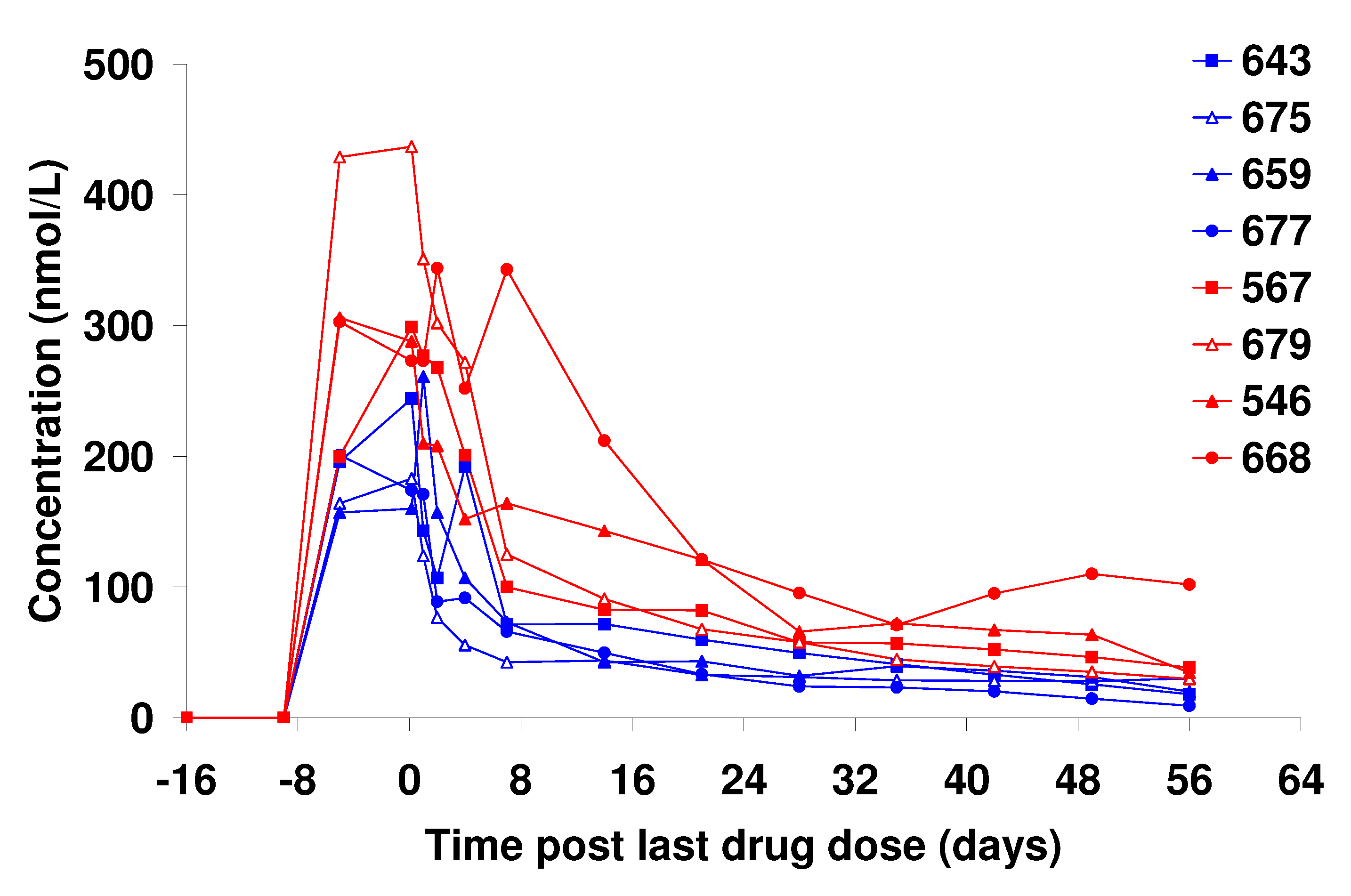

Supplement: Figure S2 — Individual plasma concentration-time profiles of DB829 following administration of DB868 to uninfected vervet monkeys. DB868 was administered orally at 10 mg/kg/day (643, 675, 659, 677; blue symbols) or 30 mg/kg/day (567, 679, 546, 668; red symbols) for 10 days day −9 to day 0 post-last drug dose. (TIF) [file pntd.0002230.s002.tif]
